# Transitioning from Acute to Chronic Pain: An Examination of Different Trajectories of Low-Back Pain

**DOI:** 10.3390/healthcare6020048

**Published:** 2018-05-17

**Authors:** Robert J. Gatchel, Kelley Bevers, John C. Licciardone, Jianzhong Su, Ying Du, Marco Brotto

**Affiliations:** 1 Department of Psychology, College of Science, The University of Texas at Arlington, 1225 West Mitchell, Box 19528, Arlington, TX 76019, USA; kelley.bevers@mavs.uta.edu; 2 University of North Texas Health Sciences Center, Fort Worth, TX 76107, USA; john.licciardone@unthsc.edu; 3 Department of Mathematics, College of Science, The University of Texas at Arlington, Arlington, TX 76019, USA; su@uta.edu (J.S.); duying@ecust.edu.cn (Y.D.); 4 College of Nursing and Health Innovations, The University of Texas at Arlington, Arlington, TX 76010, USA; marco.brotto@uta.edu

**Keywords:** pain, low-back pain, chronic, acute, subacute, pain trajectories

## Abstract

Traditionally, there has been a widely accepted notion that the transition from acute to chronic pain follows a linear trajectory, where an injury leads to acute episodes, subacute stages, and progresses to a chronic pain condition. However, it appears that pain progression is much more complicated and individualized than this original unsupported assumption. It is now becoming apparent that, while this linear progression may occur, it is not the only path that pain, specifically low-back pain, follows. It is clear there is a definite need to evaluate how low-back pain trajectories are classified and, subsequently, how we can more effectively intervene during these progression stages. In order to better understand and manage pain conditions, we must examine the different pain trajectories, and develop a standard by which to use these classifications, so that clinicians can better identify and predict patient-needs and customize treatments for maximum efficacy. The present article examines the most recent trajectory research, and highlights the importance of developing a broader model for patient evaluation.

## 1. Introduction

After evaluating the lack of attention to the problem of pain and pain care in the United States, the Institute of Medicine (IOM) highlighted the importance of addressing this prevalent and costly issue [1]. In response, the National Pain Strategy (NPS) was formulated and released in 2016, with the intention to improve pain care in the US. This then stimulated the formation of the Interagency Pain Response Coordinating Committee (IPRCC; https://iprcc.nih.gov), with the charge to develop and prioritize specific research recommendations in order to advance the NPS agenda. Two of the present authors (Gatchel and Licciardone) were members of the IPRCC. One area of importance identified was to “*…evaluate the time-course of pain from an initial acute episode, to more serious episodic “flare-ups”, and then to chronic states of pain and the resulting costly disability and treatment needs*”.

Currently, not enough is known about the different trajectories and exact timing of when pain occurs or becomes chronic [2]. Such important information will lead to a better pairing of interventions that target patients with pain at critical “windows of opportunity” that would have the most important impact on healthcare and economic resources. In the past, there has been the general assumption, while not empirically supported, that there was a natural linear transition from acute, to subacute, and then to chronic pain (e.g., [3,4,5]). However, as noted earlier by van Tulder and colleagues (2002) when evaluating low-back pain (LBP), “*…there is a need to revise our views regarding the course of low-back pain*” (p. 761). Others have come to the same conclusion (e.g., [6,7]).

The purpose of the present investigation was to review the various trajectories that have been delineated by different clinical research groups, focusing on low-back pain (LBP) for a number of important reasons. First, as highlighted in the IOM report “Relieving Pain in America” [8], musculoskeletal pain is the most common single type of chronic pain; chronic LBP (CLBP) is the most prevalent in this category. Indeed, the economic burden of LBP is quite large, and continues to grow in the US. Total healthcare costs for LBP, combining direct and indirect costs, have been estimated to amount to roughly $100 billion annually [8,9]. Moreover, as reviewed by Luo and colleagues [10], approximately 80% of Americans will experience at least one episode of back pain during their lifetimes, and 15–20% report back pain at some time in a one-year period. Kongsted and colleagues [11] point out that, previously, LBP was categorized as acute or chronic, where acute sufferers experienced one or more events of *unrelated* pain or a continuous pain state.

The second major reason for focusing on LBP is that the National Health Institutes Research Task Force (NIH RTF; [12]) concluded that one very important obstacle which hampered LBP investigations in the past was the lack of an agreed-upon case definition of CLBP. The task force defined CLBP as “*…a back pain problem that has persisted at least 3 months and has resulted in pain on at least half of the days in the past 6 months. A human drawing would illustrate the region defined as the low-back, indicating the space between the lower posterior margin of the rib cage and the horizontal gluteal fold*” [12] (p. 573). We hope that, with this new case definition, there will be more homogeneity in how CLBP is defined.

## 2. Methods

**Figure 1:**
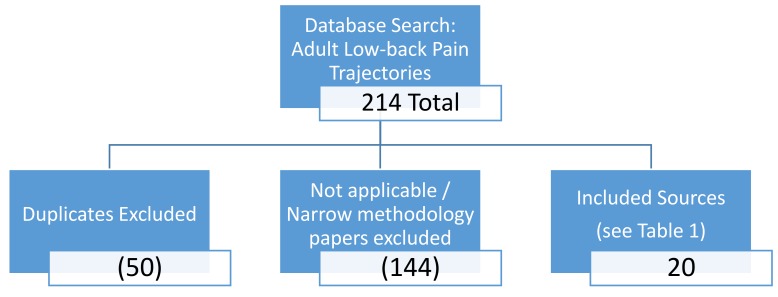
Flow Chart of Background Literature Selection.

Trajectory research papers were retrieved through a series of searches in databases, such as PubMed, psycINFO, Interagency Pain Research Portfolio (IPRP), Science Direct, and Wiley Online Library. Keywords, such as “trajectory”, “pain trajectories”, “adult pain trajectories”, “adult low-back pain”, “low-back pain trajectories”, and “acute chronic transition” were used to search each database for relevant pain trajectory research. As an example, the term “adult low-back pain trajectories” included in titles, abstracts, and keywords yielded: 47 results in PubMed; 0 results in psycINFO; 0 results in IPRP; 163 results in Science Direct; and 3 in Wiley Online Library. Additionally, key authors involved in trajectory research were identified, and searched for other relevant projects. Furthermore, references used in these works were also searched to find additional content including the articles regarding children and adolescents. Of the articles found, approximately 20 were deemed appropriate, including identified trajectories in original research and review articles (see Figure 1). Research specifically targeted towards cancer and pain, as well as duplicates, were excluded. Table 1 presents the various studies, with identified pain trajectories, that were included in the present investigation. Due to the limited amount of research on this subject, all study designs regarding chronic pain patients and all published relevant trajectory research were considered for inclusion in this analysis.

## 3. Results

Axen and Leboeuf-Yde [13] highlighted the fact that a paradigm shift is greatly needed in our thinking that LBP is usually a simple self-limiting condition, to a broader conceptualization of it as a persistent or recurrent problem. They summarized studies that revealed different trajectories: three persistent courses; a more fluctuating course; and an episodic course. These authors go on to state that, in the case of LBP, “*…the different trajectories that people experience over their lifetime have not been mapped out nor is the clinical importance of various lifetime trajectories known*” (p. 604). Additionally, Deyo and colleagues [14] evaluated the trajectories of symptoms and function in a cohort of 3929 elderly adults with LBP. They found that, although most patients remained relatively stable over one year, latent-class analysis identified other subgroups related to functional improvement and pain improvement. For functional improvement, there were the following subgroups: stable low disability; stable low-moderate disability; stable moderate-high disability; stable high disability; and functional recovery. For pain improvement, there were six distinct trajectories: four were essentially stable showing minimal improvement (but with varied starting points); and two trajectories (moderate-pain recovery and severe-pain recovery) that displayed substantial improvement over the one-year period. The latter patients displayed a shorter-duration of pain, with higher confidence in their recovery, and experienced fewer comorbidities. Based on the above results, these authors again identified different trajectories for LBP, and concluded that “*Better understanding of variability in recovery… may help target patients for more intensive interventions, plan resource use, and design clinical studies of more homogenous patient groups*” (p. 1352).

Other recent analyses have attempted to further designate subgroups by pain intensity measures into more defined trajectory patterns and single-stage or two-stage intensity and disability classifications [15,16]. Kongsted and colleagues [15] conducted a supplementary analysis and produced multiple latent class analysis models with 5–12 groups. The authors eventually decided on an 8-group model including the following groups: recovery; late recovery; slow improvement; mild episodic; improvement with relapse; moderate ongoing daily; moderate ongoing non-daily; and severe ongoing. Of the 1077 participants, the episodic condition was most common, followed by a fluctuating pattern. The researchers measured weekly pain intensity and variation measures, with a mean age of 45 years old, although a range of participants from 18 to 65 was also recruited [15]. Nielsen and colleagues [16] looked at the trajectories, and proposed a single stage or two-stage classification, arguing this methodology would have more clinical significance. More research using their techniques and analyses should be conducted to determine if this method could be a better form of trajectory classification for the precise determination and treatment approach of LBP patients. An additional differing approach, aimed to focus on physical functioning as an overall quality-of-life measure, has been used which still identified three distinct trajectories: low physical function; moderate physical function; and high physical function [17]. Consistent with previous literature, worse physical functioning was associated with higher age, more depressive symptoms, more avoidance behaviors, and lower perceived self-efficacy [17]. Enthoven and colleagues [18] also identified three distinct groups: low pain; intermediate pain; and high pain. Six hundred and seventy five participants were followed over the course of three years, with: baseline; six-week; three-month; six-month; nine-month; one-year; two-year; and three-year follow up visits. The authors do point out that the yearly visits after the one-year mark is a limitation, and suggest a more frequent schedule is needed [18]. A three-month period, as done in the beginning of their design, may be a useful evaluation period as a great deal can happen in a year’s time, particularly the fluctuation and/or re-occurrence of pain episodes or subsequent injury.

Macedo and colleagues [19] further highlighted the need to distinguish fluctuating patterns more comprehensively. Their study followed a cohort over a one-year period, with the requirement that participants had experienced persistent pain for at least three-months prior to the start of the study. They measured pain parameters at baseline, once monthly, and at the one-year follow-up. Fluctuating patterns were found in two of their three identified clusters, and significant differences in disability between those with fluctuating and those with non-fluctuating pain patterns were found. Chen and colleagues [20] found five trajectories, with those within the fluctuating trajectory returning to work most quickly, and spending the shortest time on disability. Furthermore, they identified the fluctuating and continuous high-pain trajectories to encompass the largest groups in their sample. However, it is unclear how the fluctuating patients were doing after the one-year mark. In yet another study, Downie and colleagues [21] focused on evaluating an acute episode of LBP, by following 1585 patients (average age of 45 years) from a randomized controlled trial in Australia for an investigational medication over 12-weeks. They assessed patients at baseline, and 1-, 2-, 4-, and 12-weeks thereafter. These researchers observed a best-fit model with five clusters, including recovery, persistent, and fluctuating trajectories. Combining the previously identified patterns would result in a better clinical usage of the trajectories to predict and manage LBP. Finally, another study found distinct differences in the fluctuating trajectories when measured weekly, rather than monthly [22]. Kongsted and colleagues [22] evaluated both frequency and intensity, as well as specific patterns in speed of improvement, degree of the fluctuation, and relapse rate. With a more intensive analysis, this study resulted in 12 distinctive models for analysis. Additionally, in the most recent cohort study in the literature, Chen and colleagues [23] analyzed 281 patients ages 18–60 years at baseline, 6-months, and 5-years via questionnaire collection regarding psychosocial variables, in addition to pain intensity, duration, and physical disability. Participants were designated into pre-defined clusters: no or occasional mild; persistent mild; fluctuating; and persistent severe. These were confirmed by cluster analysis. Results revealed that trajectories can be identified during primary clinical consultations, and these four trajectory patterns were generalizable with good external validity [23]. Patients’ perceptions about their pain were also examined. The authors argue that this factor is critical, because attitudes and beliefs can be modified, leading to potential improvements in pain patients’ management and recovery; such patient attitudes should be evaluated in future studies, possibly following educational programs [23].

Until now, the trajectory review has focused solely on adults in the literature, but chronic pain is prevalent in all age groups, including children and adolescents. Particularly concerning LBP, there is a definitive need to examine all age group trajectories for treatment implications. A recent study by Simons and colleagues [24] examined chronic pain and functional disability at five time points over the course of one year. They found three groups emerging for chronic pain: early treatment responders; late treatment responders; and non-responders. While they did not find psychosocial differences between early and late responders, they did find higher pain levels, older age, higher anxiety, and fewer social difficulties to be characteristic of non-responders vs. responders [24]. Additionally, Banez and colleagues [25] found four groups: stable long-term improvement; late improvement; short-term improvement; and non-responders. The authors did not find any age differences, but they did also find a relationship between response to treatment and anxiety, such that those who did not respond or responded short-term had higher anxiety levels [25]. Another study by Palermo and colleagues [26] used online cognitive behavioral therapy (CBT) as part of a randomized controlled trial (RCT) for pain treatment and found a four model solution: no or little improvement; moderate improvement; substantial improvement; and initial responders with relapse. Further work is needed to distinguish any critical age differences, possibly accounting for anxiety and resilience, as they may also fluctuate with age.

Levels of Evidence for each of the aforementioned reviewed studies are displayed in Table 2. It should be noted that, for each study, a Levels of Evidence metric (adapted from material published by the Centre for Evidence-Based Medicine, Oxford, UK: www.cebm.net) was used to rate the quality of the studies, with decreasing levels representing poorer-quality studies. It includes Levels I–V where, for example, Level I is a high-quality prospective study, and Level II is a retrospective study. Two of the authors (RJ and KB) independently rated these studies for inter-rater reliability. If there were any discrepancies, these authors met to make a final decision as to the appropriate level to give a study. As can be seen, the vast majority of studies in the present review were Level II studies, representing good-quality retrospective investigations. Thus, these reviewed studies are clearly relevant and robust in revealing the potentially different trajectories of pain and disability that will need to be considered in future studies.

## 4. Discussion

For many, LBP problems do not resolve completely; instead, they are often characterized by “flare-ups” or recurring episodes, as well as residual pain problems [27]. In addition, of those who still reported pain after 3-months, less than 10% experienced a decrease at 1-year. Thus, once one experiences an episode of LBP, it is likely to continue to be bothersome, and often impairs functioning [28]. As a consequence, LBP rates are the number-one global health burden (of 291 conditions studied) in terms of the number of years living with a disability [29]. This is in sharp contrast to earlier traditional views that back pain will spontaneously get better over time. The effectiveness of more intensive pain interventions with both physical activity and medication has been noted previously [30,31]. However, we have also been in the midst of an opioid epidemic, causing extremes to emerge in the medical community, such as overuse of medication therapies or hesitation from clinicians and patients alike. Furthermore, it has been established that interdisciplinary treatment strategies are generally most effective, allowing for the customization of a treatment plan for the individual patient (e.g., [32]). Understanding how to identify pain trajectories would be of great importance in tailoring treatment strategies to patients experiencing pain, both at the acute and chronic stages. Indeed, considering that the investigations reviewed in the present study are clearly relevant and robust for potentially determining a “window of opportunity” for best administering an intervention, it would be advantageous to further study such trajectories with larger patient sample-sizes in order to possibly condense the number of trajectories to consider in treatment-planning efforts for early intervention. For example, it would be clinically useful to determine if the length of time in recovery phases, before relapse occurs, would predict both relapse itself, as well as if a patient will transition from a fluctuating trajectory to a more persistent one. Relatedly, does the intensity of the fluctuation determine subsequent relapses? Such fluctuations can be rather small or intense. Does the severity increase the likelihood of transitioning to a more consistent state of pain?

Another important consideration would be to delve deeper into consistent biopsychosocial predictors, not only of CLBP, but of relapse and recovery as well. The association of pain occurrence and increasing age, depression, and confidence in recovery is evident [21,22,33]. It is known that there is a strong bidirectional relationship with variables such as depression and pain, and there tends to be an association with increasing age and the occurrence of pain conditions. A link between increasing age and increasing pain severity also exists [14,20,34]. In addition, the experience of pain is reportedly different between sexes, and many psychosocial factors affect the process of managing pain conditions, such as activity limitation, work participation, history of back or leg pain, anxiety, and catastrophizing [35,36]. Kongsted and colleagues [22] reiterate that, of the above-listed variables, there were observed relationships between pain patients and these measures, with the exception of gender; and mixed-evidence concerning sleep disturbances and patient education level. They further note that these variables were not used to group patients into trajectory patterns. It would be useful to determine what biopsychosocial configurations will affect the trajectory a patient follows long-term.

However, the relationships between trajectories and these variables are still unclear. Are these variables responsible for maintaining an individual trajectory, and to what extent? Kongsted and colleagues [22] reviewed the then-current landscape of trajectory research, finding that researchers had uncovered a few consistent patterns across cohorts, including: recovery (in all but [34]); persistent; and fluctuating trajectories. Two to 12 trajectories exist, with 4–5 optimal for categorization. This present article included a variety of studies, with ranges between 3-months and 5-years in duration, and varied follow-up points from weekly, monthly or a few times, over the course of the study. It is interesting that, even with the variance in design, similar trajectories among pain patients are found in the literature. It should also be noted that most authors highlighted the fact that such research is lacking in both children and older adults.

While it is known that these episodic and continuous trajectories do exist, it has become increasingly clear how many LBP patients fluctuate far beyond these simply-defined parameters. Furthermore, it is clear that individual-experiences differ greatly, and varieties of trajectory patterns exist. These trajectories sometimes overlap (see Table 1), with approximately four-to-five distinct subsets [22,23,33,37]. For example, Axen and colleagues [37] studied their sample for six-months, and identified four clusters: stable; fast improvement; gradual improvement; and slow improvement. Those in the fast-improvement trajectory experienced the fewest days of pain interference, while those in the slow-improvement trajectory experienced the most interference. The gradual-improvement cohort was defined as a “typical patient”, and their characteristics closely mimicked those of patients most often seeking treatment. Recent findings from Chen and colleagues [23] also suggest that the four trajectories defined in their study possess good external validity from a baseline visit through 5-years, allowing for better fit to classify pain patients. However, these patterns should be analyzed further in order to reach a more general consensus in the classification of the trajectory subsets.

Fortunately, there are currently available cutting-edge methods, such as daily electronic diaries using smart phones (e.g., [38,39,40,41]) to systematically track patients, from the acute LBP injury and over the following one-year periods, using an inception-cohort study design. Finally, once the trajectories are delineated, they will also provide guidance on biomarkers that may be used to better understand the underlying neuroscientific-transition mechanisms involved in order to help prevent CLBP.

## 5. Summary and Conclusions

We know that several different pain trajectories exist in LBP patients, but a comprehensive understanding of how these patients follow a defined set of trajectories is not yet totally understood. Further delineation of these trajectories will lead to better early intervention strategies, tailored treatment plans, and better outcomes for patients. Better understanding of the trajectories will also provide insight into the mechanisms of pain, and the pathways from acute injury to chronic conditions. Studying the projections in older populations with LBP will be particularly clinically useful considering the prevalence of this type of pain, and associated costs, in older adults. It is important to continue to identify risk factors for developing LBP, such as older age, depression, higher perceived pain intensity, history of pain, and catastrophizing behaviors. Understanding more about pain is of great importance, considering that the majority of the population will experience more than a single incidence of pain in their lifetimes. It is also important to note that research is lacking in all age groups, and further studies involving pediatric chronic pain could contribute to the field as well, by establishing how patterns may fluctuate or remain stable over a lifetime. Additionally, the prevalence of LBP and the aging population highlight the important role of defining the pain trajectories in these particular populations. Determination of how to better intervene, particularly for those who are on trajectories to develop chronic conditions, and in ways that maximize benefits to the individual, is of critical importance. Moreover, with the increases in the opioid crisis, the significant-aging population, and the high prevalence of pain in the general population, it is crucial to identify risk factors, and design effective and lasting interventions. It is possible that identifying the pain trajectory a patient is following could allow clinicians to best predict ideal strategies to maximize the individuals’ recovery. This is not a novice conceptualization:

“…the trajectory of your life is no longer just one straight path to an eventuality, but is instead one path to many, on one ever-branching tree of possibilities.”

Kevin Michel, from “Many Worlds of Interpretation of Quantum Mechanics”

## Figures and Tables

**Table 1:** Trajectory Classification by Reference Study.

Authors	Year	Timeline	*N*		Identified Trajectories	Design
Dunn, Jordan, and Croft	2006	Monthly for 6 months	342	4	Persistent Mild Recovering Severe Chronic Fluctuating	Observational
Chen, Hogg-Johnson and Smith	2007	4/10/16/52 weeks	678	5	Continuous High Fluctuating Large Reduction Moderate Reduction Increasing	Observational
Kongsted and Leboeuf-Yde	2009	Weekly for 18 weeks	78	4	Worsened then Fluctuating Unchanged Improved then Fluctuating Improved	Observational
Tamcan, et al.	2010	Weekly for 1 year	305	4	Persistent Severe Persistent Moderate Persistent Mild Fluctuating	Observational
Axen, et al.	2011	Weekly for 6 months	176	5	Typical Stable (mild) Slow Improvement Fast Improvement Not Classified	Observational
Dunn, Campbell and Jordan	2013	Monthly for 6 months, at 7 years	155	4	No/Occasional (recovery) Persistent Mild Fluctuating Persistent Severe	Observational
Lebouef, et al.	2013	Every 2 weeks for 1 year	261	3	More or less Constant Episodic More or less Never	Observational
Banez, et al.	2014	Admission/Discharge, 1/12/24/48 months	173	4	Stable Long-term Improvement Late Improvement Short-term Improvement Non-Responders Poor Physical Function Moderate Physical Function Good Physical Function Non-Fluctuating Recovering Mild Persistent Moderate Severe Chronic Fluctuating	Observational
Hermsen, et al.	2014	Base, 6/12/18 months	407	3		Observational
Macedo, et al.	2014	Monthly for 1 year	155	5		RCT
Deyo, et al.	2015	3/6/12 months	3929	5	Stable Low Stable Low-Moderate Stable Moderate-High Stable High Recovery	Observational
Downie, et al.	2015	1/2/4/12 weeks	1585	5	Rapid Recovery Recovery by 12 weeks Incomplete Recovery Fluctuating Persistent High Pain	RCT
Kongsted, et al.	2015	Weekly for 1 year	1082	5	Mild Episodic Recovery Moderate/Severe Improvement with Relapse Slow Improvement No or Little Improvement Moderate Improvement Substantial Improvement Initial responders with Relapse	Observational
Palermo, et al.	2015	Weekly for 8 weeks	135	4		RCT
Enthoven, et al.	2016	Base, 6 weeks, 3/6/9 months, 1/2/3 years	675	3	Low Pain Intermediate Pain High Pain Chronic High Persistent Recovering Chronic Mild Persistent Recovery Late Recovery Slow Improvement Mild Episodic Improvement with Relapse Moderate Ongoing Daily Moderate Ongoing Non-Daily Severe Ongoing Severe No / Occasional Mild Persistent Mild Fluctuating Persistent Severe Early Treatment Responders Late Treatment Responders Non-Responders	Observational
Panken, et al.	2016	Baseline, 3/6/12 months	622 (299/134/195)	3		Merged 3 RCT Trials Data
Kongsted, et al.	2017	Weekly for 1 year	1271	8		Observational
Chen, et al.	2018	5 years, then 3 monthly	281	4		Observational
Simons, et al.	2018	Admission/Discharge, 1/4/12 months	253	3		Observational

**Table 2:** Levels of Evidence Ratings of Included Trajectory Studies *.

Authors	Year of Publication	Level of Evidence Rating
Dunn, Jordan and Croft	2006	II
Chen, et al.	2007	II
Kongsted and Leboeuf-Yde	2009	II
Tamcan, et al.	2010	II
Axen, et al.	2011	II
Dunn, Campbell and Jordan	2013	II
Lebouef, et al.	2013	II
Banez, et al.	2014	II
Hermsen, et al.	2014	II
Macedo, et al.	2014	II
Deyo, et al.	2015	I
Downie, et al.	2015	III
Kongsted, et al.	2015	I
Palermo, et al.	2015	I
Enthoven, et al.	2016	III
Panken, et al.	2016	II
Kongsted, et al.	2017	III
Nielsen, et al.	2017	II
Chen, et al.	2018	II
Simons, et al.	2018	II

* Levels of Evidence data were adapted from material published by the Centre for Evidence-Based Medicine, Oxford UK: www.cebm.net. Level I is the highest quality-study, followed by Levels II–V in descending order of quality.
